# A Manual of Surgical Treatment

**Published:** 1913-12

**Authors:** 


### Reviews of Books.

**A Manual of Surgical Treatment.** By Sir W. WATSON CHEYNE, Bart., C.B., and F. F. BURGHARD, M.S., F.R.C.S. New Edition, entirely revised and largely re-written with the assistance of T. P. LEGG, M.S., F.R.C.S., and ARTHUR EDMUNDS, M.S., F.R.C.S. Volume IV. Pp. xxvii, 622. London: Longmans, Green & Co. 1913. Price 21s. net.

This volume deals with affections of the jaws, nasopharynx, tongue, mouth, pharynx, œsophagus, stomach, and intestine. Thus it embraces a large proportion of that domain of surgery in which the greatest activity and most recent advances have been made. Considering the generally conservative tone of this work, the present volume represents perhaps more than others of the series the modern methods of treatment. In the injuries and diseases of the jaw there is but little new matter, but opportunity is taken to introduce the subject of intravenous etherisation and intratracheal insufflation anæsthesia in connection with the removal of jaw tumours. In the surgery of the tongue Butlin's methods of partial resection for leucoplakia and excision for cancer are closely followed. Due prominence is given to the enucleation of the tonsils, though there might be a greater insistence upon the necessity of this replacing mere tonsillotomy as the routine practice. In the description of gastrostomy the operations of Witzel, Kader-Senn and Franck are presented as though of equal merit. There is an excellent account of "exploratory laparotomy," together with remarks as to the closure of the incision. Silk is said to be the suture for all the abdominal layers, even the skin (catgut is mentioned in Figure 81 for the peritoneal layer, though silk is spoken of for this in the text). We should have thought that porous silk had nothing to recommend it in the superficial layers.

The section on intestinal obstruction is illustrated by many good new figures.

In the chapter on appendicitis, the modern view that every case should be operated upon as early as possible is stated more than once, but not quite with the earnestness of complete conviction. Such statements as "advise appendicectomy after the patient has had one attack" (p. 412), and "opinions differ as to the period at which the operation should be performed" (p. 420), afford too much justification for the common practice of "wait and see" which so often proves disastrous. We are glad, however, that the early operation is regarded as ideal, and that the appendix is always to be removed.

In dealing with hernia, a judicious selection has been made of the many operations for "radical cure." We think, however, that two points in connection with femoral hernia might be improved. First it should be stated that the ideal aimed at by a truss can never be attained, because no truss can obliterate the crural canal. Second, the radical operation by which the conjoined tendon is sutured to the pectineal fascia deserves mention. The use of silver wire filigrees for ventral hernia is advocated, without mentioning that in most cases these structures become broken and disintegrated within a year of the operation.

In the article on cancer of the rectum very brief mention is made of the abdominal method of extirpation, and here as elsewhere there might be more space devoted to definite advice as to the choice of methods suitable for different cases.

The book makes one of the most complete and valuable textbooks of reference on the subject of abdominal surgery that have appeared in recent years.

